# Hypotheses, rationale, design, and methods for prognostic evaluation in type 2 diabetic patients with angiographically normal coronary arteries. The MASS IV-DM Trial

**DOI:** 10.1186/1471-2261-10-47

**Published:** 2010-09-29

**Authors:** Whady Hueb, Neuza Lopes, Paulo R Soares, Bernard J Gersh, Eduardo Gomes Lima, Ricardo D´Oliveira Vieira, Cibele Larrosa Garzillo, Rosa Rhami Garcia, Alexandre Costa Pereira, Celia Maria Strunz, Claudio Meneguetti, Jeane Tsutsui, Jose Parga, Pedro Lemos, Alexandre Hueb, Augusto Ushida, Raul Maranhão, Dalton A Chamone, Jose AF Ramires

**Affiliations:** 1 From the Heart Institute of the University of São Paulo, São Paulo, Brazil; 2 Mayo Clinic, Rochester, Minnesota, USA

## Abstract

**Background:**

The MASS IV-DM Trial is a large project from a single institution, the Heart Institute (InCor), University of São Paulo Medical School, Brazil to study ventricular function and coronary arteries in patients with type 2 diabetes mellitus.

**Methods/Design:**

The study will enroll 600 patients with type 2 diabetes who have angiographically normal ventricular function and coronary arteries. The goal of the MASS IV-DM Trial is to achieve a long-term evaluation of the development of coronary atherosclerosis by using angiograms and coronary-artery calcium scan by electron-beam computed tomography at baseline and after 5 years of follow-up. In addition, the incidence of major cardiovascular events, the dysfunction of various organs involved in this disease, particularly microalbuminuria and renal function, will be analyzed through clinical evaluation. In addition, an effort will be made to investigate in depth the presence of major cardiovascular risk factors, especially the biochemical profile, metabolic syndrome inflammatory activity, oxidative stress, endothelial function, prothrombotic factors, and profibrinolytic and platelet activity. An evaluation will be made of the polymorphism as a determinant of disease and its possible role in the genesis of micro- and macrovascular damage.

**Discussion:**

The MASS IV-DM trial is designed to include diabetic patients with clinically suspected myocardial ischemia in whom conventional angiography shows angiographically normal coronary arteries. The result of extensive investigation including angiographic follow-up by several methods, vascular reactivity, pro-thrombotic mechanisms, genetic and biochemical studies may facilitate the understanding of so-called micro- and macrovascular disease of DM.

## Background

Diabetes mellitus is a potentially serious disease and is currently the major concern of public health officials. This disease has a prevalence of 2% to 5% in most Western countries and has grown exponentially around the world, possibly because of changing dietary habits in recent years [1-3].

Patients with type 2 diabetes often have cardiovascular risk factors that may further influence the prognosis of the disease. These risk factors compose metabolic syndrome. They include high blood pressure [4,5], dyslipidemia, and obesity, which have been reported as participating in the pathogenesis of cardiovascular events by possible action on coronary endothelial function [5]. Moreover, the high rate of urinary albumin excretion has been hypothesized as an additional indicator of endothelial dysfunction, which is associated with a higher rate of renal and cardiovascular events [6]. Other risk factors include potential age and sex [7], smoking [8], glycemic control, lipids [5,9], and hormone replacement therapies [10].

Major studies have shown that healthy endothelium inhibits adhesion of platelets and leukocytes to the vessel surface and maintains the balance of profibrinolytic and prothrombotic activity [11]. However, with the occurrence of dysfunction, the endothelium is a potential contributor to the pathogenesis of vascular disease in diabetes mellitus and atherosclerosis [12].

Under physiological conditions, there is a delicate and balanced release of relaxing factors and endothelium-derived contraction factors that apparently is imbalanced in diabetes and may contribute to the development of atherosclerosis, thus contributing to the further progression of vascular injury in different organs [13].

Hyperglycemia has been implicated as the main causal factor in the development of coronary atherosclerosis in diabetic patients. However, the mechanisms associated with this disorder are likely to be multifactorial. Among these are hypertension, abdominal obesity, and pluri-metabolic syndrome [14-16].

Besides these, we highlight the presence of decreased high-density lipoprotein and elevated low-density lipoprotein (LDL) cholesterol, high triglycerides, increased VLDL, nephropathy, altered coagulation, and platelet dysfunction. Insulin resistance, another existing condition in diabetes, has been described as responsible for the increased risk of death from coronary artery disease (CAD) [15-17].

It is assumed that the prevalence of coronary artery disease (CAD), recognized by a variety of diagnostic methods, is estimated to be 55% in individuals with diabetes. This contrasts with 2% to 4% of people with diabetes in the general population [18]. Patients with CAD without diabetes compared with those with diabetes have coronary atherosclerosis that is more advanced, with higher rates of ventricular dysfunction and cardiac events [18-20]. Additionally, the prognosis of CAD is less favorable in patients with diabetes than in their nondiabetic counterparts; mortality after infarction is higher in diabetic patients and is particularly high among women [19-22]. Often, patients with diabetes may have asymptomatic CAD. In such cases, the first sign of CAD may be the occurrence of a myocardial infarction or cardiac death [21,23]. Data reported by Haffner [24] show that patients with diabetes but without CAD have the same incidence of heart attack as nondiabetic patients with CAD. Furthermore, 79% of patients with diabetes die of cardiac complications after an acute myocardial infarction (AMI) [23]. Moreover, in patients referred to surgery, the need for new CABG or PCI is significantly higher in subjects with diabetes than in those without the disease [25]. This condition led the National Cholesterol Education Program (NCEP) to consider diabetes as a risk equivalent to that of CAD [26].

However, although the occurrence of diabetes is a major determinant of vascular lesions, a small series of retrospective studies have addressed endothelial behavior in diabetic patients with angiographically normal coronary arteries [27-29]. On the other hand, few studies have been directed at discussing in depth the occurrence of major cardiovascular events in long-term follow-up in patients with type 2 diabetes mellitus who have angiographically normal coronary arteries. So, our hypothesis is that some diabetic patients despite of absence of coronary artery based on angiographic criteria will present cardiovascular events and progression of the CAD disease but others won't along five-years of follow-up. The main aim of the MASS IV DM study is to identify what is the protective mechanism of vascular involvement on this group of diabetes patients and the correlation with cardiovascular events.

The objectives of the MASS IV-DM Trial is to compare the clinical data, laboratory profile, and angiographic evolution at baseline with that at 5-year follow-up in patients with type 2 diabetes who have angiographically normal coronary arteries.

## Methods/Design

The MASS IV-DM Trial is an institutional project to be conducted at the Heart Institute (InCor), Hospital das Clinicas, University of São Paulo, and is designed to prospectively investigate 600 type 2 diabetic patients with a clinical suspicion of coronary insufficiency and electrocardiographic evidence of myocardial ischemia who have normal coronary angiographies and ventricular function.

It is designed as a secondary prospective cohort study. Only patients with angiographically normal coronary arteries (by semi quantitative TCA equal to zero) will be included in the study. After inclusion, all patients will undergo a 320-row multidetector computed tomography (MDCT) angiography of the coronary arteries to assess the calcium score as well as the plaque characteristics if present any. Both angiography studies will be repeated after 5 years of follow-up. Ischemia induced by stress electrocardiography and associated with myocardial nuclear scintigraphy will be performed at the beginning and end of the study. Furthermore, ventricular function will be measured by echocardiography at baseline, and vascular reactivity and endothelial function will be recorded at the beginning and end of the study. Laboratory tests will include a biochemical profile of blood lipids, renal function, urinalysis, genetic polymorphism, oxidative stress, and inflammatory activity, among others. All patients will undergo an outpatient clinical evaluation every 6 months. The schedule of activities is shown in Table 1.

**Table 1:** Schedule of measurements.

	S	6	12	18	24	30	36	42	48	54	60
		m	m	m	m	m	m	m	m	m	m
History/events	X	X	X	X	X	X	X	X	X	X	X
Physical examination	X		X		X		X		X		X
Symptom assessment	X	X	X	X	X	X	X	X	X	X	X
Routine laboratory	X	X	X		X		X		X		X
Electrocardiography	X	X	X		X		X		X		X
Echocardiography	X		X		X		X		X		X
Stress MPI	X						X				X
Medications	X	X	X	X	X	X	X	X	X	X	X
Angiograms	X										X
CAC-Scan	X										X
Arterial stiffness	X										X
Resources utilization											X
Quality of life	X										X
Work status	X										X
Serious adverse events			Continuous								X

Abbreviations: S = baseline; 6m = 6 month after study entry; 12 m to 60 m denotes months after study entry. Stress MPI=Stress myocardial perfusion imaging, CAC-Scan=Coronary-artery calcium (CAC) scan

The MASS IV-DM Trial has an independent external committee that is blind to the clinical evaluation and will monitor the research. This external committee will analyze all clinical events including myocardial infarction, cerebrovascular events, and others. The role of this committee will be to ensure that clinical events will be assessed uniformly.

### Patients

Diabetic patients with clinically suspected (based on presence of the CCS II/III angina) or documented myocardial ischemia based on ET will undergo coronary angiography that will be performed according to the technique of Sones or Seldinger. For the evaluation of ventricular function, patients will receive contrast in the left ventricle, and measures will be taken with the patient in the right anterior oblique position. The calculation of left ventricular ejection fraction will be made according to the Dodge formula.

For the angiographic study of the coronary arteries, 5 views of the left coronary artery and 2 of the right coronary artery will be obtained. These effects allow a clear assessment of each coronary artery, thus forming 4 pairs of perpendicular incidence suitable for biplane analysis.

Nitrates or other vasoactive drugs will be available when needed. The same sequence of arteriographic projections, field X-ray, and size of catheters will be followed in each patient and similarly in subsequent examinations. These conditions will be crucial for the accurate assessment of the artery after 5 years of follow-up.

Possible changes in the lumen of the coronary arteries will be assessed visually and quantitatively. Two experienced observers are blinded to the identity of the patient during both tests and also for the sequence of films at the beginning and end of the study.

The pair of films, the initial and final will be seen both side by side with 5 times magnification in a projector system adapted for this analysis.

To evaluate the second angiogram, 2 incidences will be selected of a good quality at the same point in the cardiac cycle as on the first angiogram of each coronary artery studied. Based on direct visual comparison of the coronary arteries, by consensus, the arteries will be rated as unchanged, definitely changed, or possibly changed. For possibly changed, a third frame will be selected for each view from each film.

### Coronary artery calcium scan

It is assumed that the presence of calcium in the wall of the coronary arteries is a strong indicator for the diagnosis of coronary artery disease [30,31]. Previous studies using electron-beam computed tomography (EBCT) have suggested that EBCT has great potential for noninvasive quantification of calcium in the coronary arteries [32,33]. Furthermore, CAD is also related to the presence of calcium with luminal narrowing of coronary arteries and correlates with clinical, angiographic, and pathology data [34,35].

However, this method of investigation was unable to precisely define the percentage of luminal narrowing based only on the presence of calcium in the wall of artery.

Recent studies have focused attention on the uncertainties of visual estimates of the percentage of luminal narrowing compared with angiography, the difficulty of making visual estimates, and therefore their reproducibility [36,37], their predictive value, changes in the flow, and also in flow reserve [38,39].

Thus, to prevent this methodological bias, we plan to evaluate the presence of calcium in the arterial wall by performing a coronary artery calcium scan using EBCT at baseline and after 5 years of follow-up and to correlate these findings with angiographic data at the beginning and end of the study. This procedure can identify possible luminal narrowing and also eliminate any methodological flaw, because it compares similar examinations at the beginning and end of the study.

### Myocardial scintigraphy

To evaluate the presence of myocardial ischemia, even in the absence of coronary epicardial vessels, all patients will undergo myocardial scintigraphy during exercise at baseline and at the end of the study. The images of myocardial scintigraphy will be acquired through the tomographic technique (SPECT - Single Photon Emission Computed Tomography), synchronized with an electrocardiographic signal (*gated*), which allows for the simultaneous assessment of myocardial perfusion and a quantitative parameter of ventricular function, by using the 99mTc-Sestamibi (2-methoxy-isobutyl-isonitrile marked with technetium-99 m) as a myocardial perfusion marker.

With the patient under resting conditions, 740 MBq of 99 mTc-Sestamibi will be endovenously administrated to the patient, and the acquisition of images will be performed 60 to 90 minutes after the injection. Near the end of the stress test, 740 MBq of 99 mTc-Sestamibi will be introduced, and the patient will be stimulated to continue exercising for at least 1 minute, if possible. After the recovery stage, data will be recorded, and the patient will be placed under the gamma-chamber detector so that images can be obtained.

The acquisition of images will be performed in a noncircular orbit, starting from a RAO 45° projection to LPO 45° projection, thus completing 180° scan for tomographic images, at 64 frames in a 64 × 64 matrix (1 detection every 2.8° with a duration of 30s), using an energy of 140 keV windows. No coefficient of spreading attenuation or correction will be used. The images will be prefiltered with a 2-dimensional Butterworth filter with order 5, cutoff frequency of 0.6 Nyquist, and reconstructed through the iterative method (5 iterations) in a Sun computer, Ultra 60 model, through the AUTO SPECT + Instill 5.0 software, according to the existing recommendations [40,41]. The analysis of ejection fraction will be performed using AUTO QUANT 4.21 software. For the quantification of ischemic myocardium, a semi-quantitative assessment will be performed of a 17-segment model (short axis and long vertical axis will be separated into 6 segments in the apical, middle, and basal sections of the short axis, and in 2 apical segments in the long vertical section), retrospectively analyzed by at least 3 experienced observers, without any knowledge of clinical, ergometric, and cinecoronariographic data [41].

At the beginning of the study and at the end of follow-up, perfusion images will be analyzed and quantified through the 5-point semi-quantitative scoring system: 0 (normal), 1 (discreet reduction in captation), 2 (moderate reduction in captation), 3 (significant reduction in captation of radioisotopes), 4 (apparent absence of detectable captation at the follow-up).

The exercise score summed (ESS) index will be acquired by summing the scores of 17 follow-ups of exercise stress test imaging [42]. The resting score summed (RSS) index will be obtained in the same way, by summing the scores of 17 follow-ups of the image of captation at each examination of rest. To estimate the reversibility of the defect, the difference in the score summed (DSS) index will be calculated through the subtraction of the ESS from the RSS index.

### Doppler echocardiography

Changes in myocardial contractility in both systole and diastole are identified by Doppler-echocardiography. Thus, to assess the presence of variation in myocardial thickness or systolic-diastolic movements even in the absence of coronary epicardial vessels, all patients will undergo echocardiography at baseline and at the end of the study.

Patients will undergo examination with commercially available 2-dimensional echocardiographic ATL equipment, with an electronic transducer of 2.5 mHz. Intermediate examinations during follow-up will not be considered. For study purposes, echocardiographic patients will be free of medications at the beginning and end of the study. Normal LV systolic function will be considered > 50%.

For the echocardiographic examination, patients will be examined while in the left lateral position with a slight rotation of the thorax, and standardized apical and parasternal approaches. Echocardiographic records follow the recommendations of the American Society of Echocardiography.

The relaxation time of the left ventricle (TRIVE) will be determined by the simultaneous recording of intracavitary blood flow on Doppler measured at the end of the closure of the aortic and mitral valve opening, corresponding to the beginning of the E-wave.

The E-wave deceleration time is calculated by the time interval between the peak E-wave and the intersection of this line with that at baseline.

The tests will be analyzed by 3 observers. The first observer will assess the measures twice and a second observer once. The third observer will be available to resolve doubts. Interobserver and intraobserver variability will be identified and corrected by consensus.

### Arterial stiffness

All patients will be evaluated for vascular stiffness at baseline and at the end of follow-up. Measurement of arterial stiffness is considered to be particularly interesting in the proposed scenario since the absence of atherosclerotic obstructions indicated by conventional angiograms, and confirmed by coronary artery calcium scan, empowers in significance prospective changes in arterial mechanical properties. It is thus conceivable that this will be a useful tool in informing the development and possible progression of atherosclerotic disease. Arterial stiffness can be assessed noninvasively by different methodologies. The measurement of aortic pulse wave velocity is generally accepted as a very reliable method since it is simple, noninvasive, and reproducible, and can be compared periodically being supported by the greatest number of epidemiological studies for its predictive value for initiation or progression of coronary artery disease.

Briefly, wave forms will be obtained transcutaneously over the radial or common carotid artery and the right femoral artery, and the time delay is measured between the feet of the 2 wave forms.

### Laboratory measurements

The following laboratory measurements will be recorded on the same day: HbA1c (agarose electrophoresis; normal value <6%; Hyrys Hydrasys, Sebia, France); serum total cholesterol, HDL cholesterol, and triglycerides (enzymatic colorimetry; Hitachi 912; Roche Diagnostic); creatinine (colorimetry; Kone Optima; Thermolab Systems, Helsinki, Finland); and urinary albumin excretion rate on a 24-h urine collection (laser immunonephelometry; BN100; Dade-Behring, Paris, France). LDL cholesterol will be calculated according to the Friedwald formula and creatinine clearance according to the Cockroft and Gault formula.

### Genetic Analysis

All patients will have genomic DNA extracted from whole blood at study enrollment. Genomic DNA will be extracted following a standard salting-out protocol. Assuming that genetic factors will be responsible, at least in part, for different outcomes of diabetic patients regarding coronary artery disease progression, we plan to use this resource for genetic risk stratification of the studied individuals. Initially, our protocol will follow previously described *loci *associated with different intermediate phenotypes of coronary artery disease pathology (ie, lipid levels, diabetes, atherosclerosis phenotypes, hypertension/blood pressure), hypothesizing that they will also modulate the risk of coronary disease progression in this particular cohort.

### Statistical analyses

All data will be expressed as means ± SD. Differences between the data of patients for clinical and biological characteristics and basal hemodynamic parameters were analyzed by the nonparametric Mann-Whitney test. Changes in hemodynamic parameters at baseline and the end of follow-up will be analyzed with the paired Student *t *test. Comparisons between coronary artery dimensions at baseline and the end of the study will be made by 2-way ANOVA with repeated measures for experimental condition factor followed by the Fisher protected least-significance difference test.

The Spearman test will be used to find the correlates of coronary endothelium-dependent vasoreactivity. Due to its skewed distribution, urinary albumin excretion rate will be log transformed in the correlation analyses. A backward linear multiple regression will be performed to find the independent predictors for endothelium-dependent coronary vasoreactivity. Statistical significance is assumed when P < 0.05.

## Discussion

The MASS IV-DM trial is a study designed to include diabetic patients with suspected myocardial ischemia whose conventional angiogram showed coronary arteries to be angiographically normal.

Even if diabetes has been implicated as the main causal factor in the development of coronary atherosclerosis, the question that arises is what are the protective mechanisms of this so-called micro- and macrovascular disease. On the other hand, the mechanisms associated with coronary artery disease are likely multifactorial, including hypertension, abdominal obesity, and plurimetabolic syndrome; diabetes plays an important role in this scenario.

Patients with CAD without diabetes compared with diabetic patients have coronary atherosclerosis that is more advanced, and rates of ventricular dysfunction and cardiac events are higher. Additionally, the prognosis of CAD is less favorable in patients with diabetes than their nondiabetic counterparts; mortality after infarction is higher in diabetic patients and is particularly high among women.

Few studies discuss in depth the occurrence of major cardiovascular events in long-term follow-up in patients with type 2 diabetes with angiographically normal coronary arteries.

The MASS IV-DM Trial is a great opportunity to address all these issues, and it will add relevant information to this field.

### Ethical Considerations

The MASS IV-DM Trial is conducted in accordance with the principles of the Declaration of Helsinki and with laws and regulations of our country. The Ethics Committee of the Heart Institute of the University of São Paulo, Brazil approved the study protocol. The attending physician obtained written informed consent from the study participants.

### Final Considerations

The MASS IV-DM trial is designed to include diabetic patients with clinically suspected myocardial ischemia in whom conventional angiography shows angiographically normal coronary arteries. The idea that diabetes mellitus is a micro- and macrovascular disease leads us to reflect on the difficulties of comprehending the pathophysiological mechanisms of the disease. The question that arises is what is the protective mechanism of vascular involvement? The result of extensive research including angiographic follow-up by several methods, vascular reactivity, pro-thrombotic mechanisms, genetic and biochemical studies may facilitate this understanding and lead to more specific studies for the prevention of so-called micro- and macrovascular disease.

## Abbreviations

MASS IV: Medical Angiplasty or Surgery Study; DM: Diabetes Melittus; LDL low-density lipoprotein cholesterol; VLDL: high triglycerides, increased; CAD: coronary artery disease; AMI: acute myocardial infarction; PCI: Percutaneous Coronary Intervention; CABG: Coronary Artery Bypass Graft; NCEP: National Cholesterol Education Program; CT: Computed tomography; MPI = Stress myocardial perfusion imaging; CAC: CAC-Scan = Coronary-artery calcium scan; EBCT: electron-beam computed tomography; SPECT: Single Photon Emission Computed Tomography; MBq Mega Becquerel; Tc: technetium; RAO: Right anterior oblique; LPO: Left posterior oblique; keV: Kilo eletrovolts; ESS: exercise score summed; RSS: resting score summed; DSS: difference in the score summed; mHz: mili hertz; TRIVE: relaxation time of the left ventricle; HbA1c: haemoglobin A1C; DNA: Deoxyribonucleic acid

## Competing interests

None of the authors of the MASS IV-DM Trial has a financial or any other relation that would pose a conflict of interest.

## Authors' contributions

Each of the authors has made substantial contributions either to the conception and design of the study or to the drafting of this article and critical revision for this important intellectual content. Specifically, WH is the Principal Investigator of the study described in the manuscript; WH, RM, DC, ACP, PL, and JAFR actively participated in designing and performing the research. Additionally, CM, JT, and JP performed the examination procedures. WH, ACP, RR, and NHML are following all the patients during the follow-up clinical visits. Finally, NHML, ACP, RM, and DC planned the Ancillaries Studies. All authors participated in drafting and revising the manuscript, and all authors read and approved the final manuscript.

## Pre-publication history

The pre-publication history for this paper can be accessed here:

http://www.biomedcentral.com/1471-2261/10/47/prepub
